# Minimal important change and other measurement properties of the Oxford Elbow Score and the *Quick* Disabilities of the Arm, Shoulder, and Hand in patients with a simple elbow dislocation; validation study alongside the multicenter FuncSiE trial

**DOI:** 10.1371/journal.pone.0182557

**Published:** 2017-09-08

**Authors:** Gijs I. T. Iordens, Dennis Den Hartog, Wim E. Tuinebreijer, Denise Eygendaal, Niels W. L. Schep, Michael H. J. Verhofstad, Esther M. M. Van Lieshout, on behalf of FuncSiE Trial Investigators

**Affiliations:** 1 Trauma Research Unit, Department of Surgery, Erasmus MC, University Medical Center Rotterdam, Rotterdam, The Netherlands; 2 Department of Orthopaedic Surgery, Upper Limb Unit, Amphia Hospital, Breda, The Netherlands; 3 Trauma Unit, Department of Surgery, Academic Medical Center, Amsterdam, The Netherlands; Sint Antonius Ziekenhuis, NETHERLANDS

## Abstract

**Study design:**

Validation study using data from a multicenter, randomized, clinical trial (RCT).

**Objectives:**

To evaluate the reliability, validity, responsiveness, and minimal important change (MIC) of the Dutch version of the Oxford Elbow Score (OES) and the *Quick* Disabilities of the Arm, Shoulder, and Hand (*Quick-*DASH) in patients with a simple elbow dislocation.

**Background:**

Patient-reported outcome measures are increasingly important for assessing outcome following elbow injuries, both in daily practice and in clinical research. However measurement properties of the OES and *Quick*-DASH in these patients are not fully known.

**Methods:**

OES and *Quick*-DASH were completed four times until one year after trauma. Mayo Elbow Performance Index, pain (VAS), Short Form-36, and EuroQol-5D were completed for comparison. Data of a multicenter RCT (n = 100) were used. Internal consistency was determined using Cronbach’s alpha. Construct and longitudinal validity were assessed by determining hypothesized strength of correlation between scores or changes in scores, respectively, of (sub)scales. Finally, floor and ceiling effects, MIC, and smallest detectable change (SDC) were determined.

**Results:**

OES and *Quick*-DASH demonstrated adequate internal consistency (Cronbach α, 0.882 and 0.886, respectively). Construct validity and longitudinal validity of both scales were supported by >75% correctly hypothesized correlations. MIC and SDC were 8.2 and 12.0 point for OES, respectively. For *Quick*-DASH, these values were 11.7 and 25.0, respectively.

**Conclusions:**

OES and *Quick*-DASH are reliable, valid, and responsive instruments for evaluating elbow-related quality of life. The anchor-based MIC was 8.2 points for OES and 11.7 for *Quick*-DASH.

## Introduction

Musculoskeletal elbow injuries may influence health and quality of life [1–3]. Physicians have traditionally been focused on objective parameters such as radiographic healing or range of motion when evaluating recovery following elbow injuries. However, patients’ own appreciation of recovery may differ from the judgment of the treating physician [4–6]. Patient-reported outcome measures (PROMs) are increasingly important for assessing outcome following elbow injuries, both in daily practice and in clinical research [7]. A multitude of such questionnaires is available for monitoring outcome over time. Region-specific questionnaires provide insight in pain and functional problems caused by specific injuries or injuries at a specific anatomic region. Generic quality of life questionnaires like the Short Form-36 (SF-36) and EuroQoL-5D (EQ-5D), on the other hand, enable comparison across populations with different injuries. Instruments should only be used if proven reliable and valid.

The best elbow-specific questionnaire currently is the Oxford Elbow Score (OES). This originally English patient-reported questionnaire measures injury-related quality of life in patients following surgery of the elbow joint [8–10]. The OES was translated into Dutch according to the guideline for Cross Cultural Adaptation of Self-Report Measures and validated for its reliability, validity, and responsiveness [11–14]. Limitations of a pilot validation study, in which the OES was compared with the DASH, were a small sample size and heterogenic population consisting of operatively and non-operatively treated patients. The OES has been shown valid and reliable for the assessment of outcome in patients with surgically treated chronic elbow pathologies [15]. However, measurement properties for patients with acute elbow injuries where full recovery is to be expected are not available.

The most often used questionnaire for upper extremity injuries is the Disability of the Arm, Shoulder, and Hand (DASH). It was designed to describe disability experienced by patients with any musculoskeletal condition of the upper extremity and to monitor change in symptoms and upper limb function over time [16]. The DASH questionnaire has been validated in patients with upper extremity musculoskeletal disorders such as rheumatoid arthritis and shoulder impingement syndrome [17–19]. The *Quick*-DASH is a shortened version of the DASH [20].

Measurement properties of the OES and *Quick*-DASH in patients with a simple elbow injury are not fully known. The Minimal Important Change (MIC), which is an important input parameter for sample size calculations in clinical studies, is not available for these scores.

The aim of the current study was to evaluate the reliability, validity, responsiveness, and minimal important change of the OES and the *Quick*-DASH in adult patients with a non-operatively treated simple elbow dislocation. The Mayo Elbow Performance Index, two general health-related quality of life instruments and subscales (*i*.*e*., Short Form-36 and EuroQoL-5D), and pain measured with a Visual Analog Scale were used for comparison.

## Materials and methods

### Study data

Data of a multicenter randomized clinical trial comparing early functional treatment with plaster immobilization in patients after a simple elbow dislocation (FuncSiE-trial) were used. The trial is registered at the Netherlands Trial Register (NTR2025). The results of this study and the study protocol are published elsewhere [21, 22]. The study was approved by the Medical Research Ethics Committees or Local Ethics Boards of all participating centers. The study was approved by the Medical Research Ethics Committees of Erasmus MC (registration number MEC-2009-239) and Local Ethics Boards of all participating centers (i.e. Red Cross Hospital (Beverwijk), Bronovo Hospital (The Hague), Westfriesgasthuis (Hoorn), Reinier de Graaf Gasthuis (Delft), Slotervaart Hospital (Amsterdam), Onze Lieve Vrouwe Gasthuis (Amsterdam), Medical Center Haaglanden (The Hague), Zaans Medical Center (Zaandam), Academic Medical Center (Amsterdam), Deventer Hospital (Deventer), Maasstad Hospital (Rotterdam), Leiden University Medical Center (Leiden), Hospital Rivierenland (Tiel), Elkerliek Hospital (Helmond), Flevo Hospital (Almere), Medical Center Alkmaar (Alkmaar), Groene Hart Hospital (Gouda), Haga Hospital (The Hague), Diakonessenhuis (Utrecht), Amphia Hospital (Breda), Admiraal de Ruyter Hospital (Goes)).

### Patients

Patients were recruited from August 25, 2009 until September 18, 2012. Inclusion criteria were 1) age of 18 years or older; 2) a simple elbow dislocation with successful close reduction; and 3) written informed consent. Exclusion criteria were 1) polytraumatized patients; 2) recurrent or open dislocation; 3) additional traumatic injuries of the affected arm; 4) surgical intervention; 5) impaired elbow function prior to trauma (*i*.*e*., stiff or painful elbow or neurological disorder); 6) previous operations or fractures involving the elbow; and 7) expected problems with completing follow-up (*e*.*g*., insufficient comprehension of the Dutch language). Baseline characteristics were gender, age, affected side, and hand dominance. Patients completed a set of questionnaires during outpatient visits at six weeks and at three, six, and 12 months after randomization.

### Questionnaires

The OES is a 12-item, three domain (elbow function, pain and social-psychological; 4 items each) questionnaire, reflecting injury-related quality of life. Each domain is transformed into a 100-point metric scale with higher score representing better outcome [15]. The same accounts for the total score. The original version was validated against the DASH [9, 15]. They showed a generally better performance for the OES than for the DASH in patients with elbow pathologies. The OES is available in several languages, and all validation studies to date were done in comparison with the DASH [23, 24]. The OES was translated from English into Dutch in compliance with translation guidelines [10, 12–14]. A pilot validation study was done in comparison with the DASH and confirmed sufficient reliability and validity in a heterogeneous group of patients with elbow pathologies [13]. Permission for the use of the OES for this study was obtained from Oxford and Isis Outcomes, part of Isis Innovation Limited (http://www.isis-innovation.com/).

The DASH is the most used questionnaire for disorders across the entire upper extremity. Validated versions are available in a multitude of languages, including Dutch. Sufficient validity, reliability, and responsiveness of the DASH has been shown for disorders across the entire upper extremity [25]. The *Quick-*DASH contains 11 items (scored 1–5) and reflects both function and pain in persons with musculoskeletal disorders of the upper extremity. To be able to calculate a score, at least 10 of the 11 items must be completed. The score is calculated using the formula: ((sum of all item/number of questions answered)-1)*11). The overall score ranges from 0 to 100 points with higher score representing greater disability [18, 25]. Reliability and validity were confirmed for the original version of the *Quick*-DASH compared with the DASH [20].

The Mayo Elbow Performance Index (MEPI) consists of four domains: pain (one item, maximum score 45 points), range of motion (20 points), stability (one item, 10 points), and function (5 items, 5 points each). Each domain is transformed into a 100-point scale with higher score representing better outcome [26].

A Visual Analog Scale (VAS) was used to measure the level of pain. The ends of the 100-mm horizontal line showed the word descriptors ‘no pain’ at 0 mm and ‘worst pain imaginable’ at 100 mm) [27].

The SF-36 is a validated 36-item health survey. It represent eight health domains (physical functioning (PF; ten items), role limitations due to physical health (RP; four items), bodily pain (BP; two items), and general health perceptions (GH; five items), vitality, energy, or fatigue (VT; four items), social functioning (SF; two items), role limitations due to emotional problems (RE; three items), and general mental health (MH; five items) that are combined into a physical and a mental component summary (PCS and MCS, respectively). The score ranges from 0–100 with higher scores representing higher quality of life. The scores are converted and compared with the norms for the general population of the United States [28]. A validated Dutch version is available [29].

The EQ-5D-3L is a validated instrument for measuring health-related quality of life. The EQ-5D utility score (EQ-US) ranges from 0 to 1 and is determined from five 1-item domains: mobility, self-care, usual activities, pain/discomfort, and anxiety/depression. In addition, the individual’s rating of his/her quality of life state is recorded by means of a standard Visual Analog Scale (EQ-VAS), which ranges from 0 to 100. Higher scores represent better health-related quality of life [30, 31]. A validated Dutch version is available [30].

### Statistical analysis

#### Basic statistics

Analyses were performed using the Statistical Package for the Social Sciences (SPSS) version 21. The receiver operating characteristic (ROC) curve and Youden index were analyzed using MedCalc 14.10.2 software (MedCalc Software, Ostend, Belgium). Data are reported in compliance with the COnsensus-based Standards for the selection of health Measurement INstruments (COSMIN) guidelines. Since raw data for individual items were analyzed, missing data were not imputed. Descriptive statistics was used in order to describe the main characteristics of the study participants. Measurement properties of the OES and *Quick*-DASH (sub)scales were determined by comparing these (sub)scales with the VAS (for pain) MEPI, SF-36, and EQ-5D.

#### Reliability

Internal consistency is a measure of the extent to which items in a (sub)scale are correlated (homogeneous), thus measuring the same concept [4]. For each (sub)scale, correlation between the items was calculated using Cronbach’s alpha. Internal consistency can be considered sufficient if the Cronbach’s alpha value is between 0.70 and 0.95, provided that the scale is unidimensional [4]. The six week data were used, since the largest heterogeneity in the degree of recovery and consequently the largest variability in scores were expected at that time.

#### Construct validity

Validity is the degree to which a questionnaire measures the construct it is supposed to measure. As there was no gold standard in the current study, the validity of the OES was expressed in terms of the construct validity. Construct validity represents the extent to which scores on a specific questionnaire relate to other measures in a way that is in agreement with prior theoretically derived hypotheses concerning the concepts that are being measured [4]. The six weeks data were used. Construct validity of the OES was assessed by determining the correlation of the OES (sub)scales with (sub)scales of the *Quick-*DASH, MEPI, SF-36, and EQ-5D. Similar procedures were followed for the *Quick*-DASH. Since all data deviated from a Normal distribution (*i*.*e*., Shapiro-Wilk test had a p<0.05 for each (sub)scale), Spearman’s Rho (rank correlation) coefficients (r) were determined. Strengths of correlation was categorized as high (r>0.6), moderate (0.3<r<0.6), or low (r<0.3) [32]. Construct validity was considered sufficient if at least 75% of the results were in line with the predefined hypotheses in a (sub)sample of at least 50 patients [4]. Predefined hypotheses are shown in S1 Table and were made in consensus between three authors (GITI, DDH, and EMMVL).

#### Responsiveness

Responsiveness refers to the ability of a questionnaire to detect clinically important changes over time [4]. Longitudinal validity can be considered to be a measure of responsiveness. Longitudinal validity refers to the extent to which change in one measurement instrument relates to corresponding change in a reference measure [33]. Analogous to construct validity, longitudinal validity was assessed by testing predefined hypotheses about expected correlations between changes in OES and *Quick*-DASH (sub)scales and changes in all other (sub)scales. Change scores were calculated as the difference in score at six weeks (which is the first time all instruments were administered) and the final score at 12 months follow-up. Since all change scores deviated from a Normal deviation, Spearman correlation coefficients were calculated. Predefined hypotheses are shown in S1 Table. Longitudinal validity was considered sufficient if at least 75% of the results were in line with the predefined hypotheses in a (sub)sample of at least 50 patients [4].

The effect size (ES) and standardized response mean (SRM) were determined as measures of the magnitude over time. The ES was calculated by dividing the mean change in score between two time points (*i*.*e*., score at 12 months–score at six weeks) by the standard deviation of the first measurement [34]. The SRM was calculated by dividing the mean change in score between two time points (*i*.*e*., score at 12 months–score at six weeks) divided by the standard deviation of this change [34]. These effect estimates were interpreted according to Cohen; a value of 0.2–0.4 is considered a small, 0.5–0.7 a moderate, and ≥ 0.8 a large effect [32]. Large effect sizes were expected a priori, since at six weeks patients were expected to have functional limitations, whereas at 12 months full recovery was expected for most patients.

#### Floor and ceiling effects

Floor and ceiling effects are present if more than 15% of the study population rates the lowest (floor effect) or highest (ceiling effect) possible score on any questionnaire (sub)scale [35]. In the presence of floor and ceiling effects, items might be missing from the upper or lower ends of the scale, reducing content validity. Likewise, patients with the highest or lowest scores cannot be distinguished from one another, indicating limited reliability [4]. Floor and ceiling effect were determined for each follow-up moment separately.

#### Minimal important change and smallest detectable change

The minimal important change (MIC) is defined as the smallest measurable change in outcome score that is perceived as significant by patients [36]. An anchor-based method was used as this gives a better indication of the importance of the observed change to the patient [4]. In addition to the questionnaires patients were asked to complete a transition item (anchor question) evaluating their perception of change in the general condition of their affected elbow. The question was: How would you judge the condition of your elbow, compared with the last time you completed this questionnaire? The item scored from 1 ‘completely recovered‘ through 2 ‘much better’, 3 ‘slightly better’, 4 ‘no change’, 5 ‘slightly worse’, 6 ‘much worse’, or 7 ‘worse than ever’. The anchor or transition item was judged as adequate if a Spearman’s rank correlation between the anchor and the change score of the questionnaire was > 0.29 [37]. The corresponding change score (score at previous follow-up subtracted from the score at time of completion of the transition item) for patients who answered the transition item as ‘slightly better’ can be considered the MIC [38].

As an alternative, MIC was also calculated for the total scores by plotting the receiver operating characteristic (ROC) curve of the change in score for patients who scored ‘slightly better’ on the transition item versus patients who scored ‘no change’. The optimal ROC cutoff point (*i*.*e*., the associated criterion of the Youden index) reflects the MIC. This MIC is shown with its 95% confidence interval (CI) after bootstrapping (1000 replicates and 900 random-number seeds).

In addition to the MIC, the Smallest Detectable Change (SDC) was determined. SDC is defined as the smallest intra-personal change in score that represents (with p<0.05) a ‘real’ difference above measurement error [7]. As patients were assumed to be stable in the interim period, this was based on the change scores of patients who answered ‘no change’ on the transition item. First, the SEM was calculated by dividing the standard deviation of the mean difference between both measurements (SD_change)_ by the square root of two [39]. SEM can be considered as a measure of absolute measurement error [4]. For the individual patient, the SDC was calculated as 1.96 x square root of 2 x SEM (herein, SEM = SD_change_ / square root of 2) [4]. Ideally, for evaluative purposes, the SDC should be smaller than the MIC [4].

## Results

One hundred patients were included, of which 48 were treated with early mobilization and 52 with plaster immobilization for three weeks. The median age was 46 year (P_25_-P_75_ 32–59) and 42 patients were male. The dislocation involved the right arm in 53 patients, and the dominant side was affected in 46 patients. One patient was lost to follow-up and six missed one follow-up visit.

### Reliability

The Cronbach’s alpha of OES total scale and all subscales ranged from 0.783 to 0.882. Cronbach’s alpha of the *Quick*-DASH was 0.886. This represents adequate internal consistency for both (sub)scales (Table 1). Internal consistency was also adequate for SF-36 (sub)scales (Cronbach’s alpha between 0.747 and 0.974), apart for the Bodily Pain (BP) subscale, which had a Cronbach’s alpha of 0.664. Cronbach’s alpha of the EQ-5D US and MEPI did not reach the Cronbach’s alpha threshold value of 0.70, but since these scales are not unidimensional, these values should be interpreted carefully. Internal consistency of the VAS and ED-5D VAS could not be determined, as they consist of one item only.

**Table 1:** Internal consistency of the instruments used in patients with a simple elbow dislocation.

(Sub)scale		N	Number of items	Cronbach’s alpha
OES	Total	99	12	0.882*
	Pain	99	4	0.783
	Function	99	4	0.825
	Social-psychological	99	4	0.804
*Quick*-DASH	Total	99	11	0.886
MEPI	Total	99	6^†^	0.383*
	Function	99	4^†^	0.693
SF-36	Total	99	35	0.880*
	PF	99	10	0.864
	RP	99	4	0.792
	BP	99	2	0.664
	GH	99	5	0.758
	VT	99	4	0.747
	SF	99	2	0.810
	RE	99	3	0.974
	MH	99	5	0.788
SF-36	PCS	99	21	0.834
SF-36	MCS	99	14	0.853
EQ-5D	US	99	5	0.528*

*Quick*-DASH, *Quick* disabilities of the arm, shoulder, and hand; PF, physical functioning; RP, role limitations due to physical health; BP, bodily pain; GH, general health perceptions; VT, vitality, energy, or fatigue; SF, social functioning; RE, role limitations due to emotional problems; MH, general mental health; OES, Oxford elbow score; PCS, physical component summary; MCS, mental component summary; SF-36, Short Form-36; EQ-5D, EuroQoL-5D; US, utility score.

*Values should be interpreted carefully, since the total scale is not unidimensional.

^†^The item related to washing was removed from analysis, as all patients gave the same answer.

### Construct validity

Construct validity is shown in Table 2. The Spearman’s rank correlation coefficients of the OES were in line with predefined hypotheses in 35 of the 42 (83%) values, indicating sufficient construct validity. All three OES subscales have sufficient construct validity; 83% (10/12) hypotheses were confirmed. For the *Quick*-DASH and MEPI, 9 out of 12 correlations (75%) were as hypothesized, also showing sufficient construct validity.

**Table 2:** Construct validity of the instruments in patients with a simple elbow dislocation.

(Sub)scale		OES				*Quick*-DASH
		Pain	Function	Social- psychological	Total	
OES	Pain	[1.00]	0.44*	0.56*	0.76*	**-0.56**
	Function	0.44	[1.00]	0.63*	0.80*	-0.72
	Social-psychological	0.56	**0.63**	[1.00]	0.91*	-0.66
	Total	0.76	0.80	0.91	[1.00]	-0.75
*Quick*-DASH	Total	**-0.56**	-0.72	-0.66	-0.75	[1.00]
MEPI	Total	0.51	0.47	0.49	0.55	-0.64
Pain	VAS	-0.40	-0.48	-0.39	-0.48	0.57
SF-36	PF	**0.27**	0.52	0.38	0.44	-0.65
	BP	0.67	**0.48**	0.66	0.71	-0.67
	PCS	0.44	0.53	0.58	**0.61**	-0.70
	MCS	0.04	-0.06	0.09	0.09	-0.07
EQ-5D	US	0.42	0.60	0.46	0.56	**-0.70**
	VAS	0.22	0.30	**0.36**	**0.35**	**-0.39**

Spearman’s rank correlation coefficients are given for all possible combinations; N = 99 for all correlations; r>0.6 indicates high correlation, 0.3<r>0.6 moderate correlation, and r> 0.6 low correlation. Bold and underlined correlations were not hypothesized correctly. Correlations between brackets are self-correlations and are not included in the calculation of the percentage correlations predicted correctly. For the OES, the overall number of correlations is 42 (the 6 correlations given above the self-correlation that are marked with an asterisk are also mentioned in the columns to the left as reversed correlation, and are thus superfluous).

*Quick*-DASH, *Quick* disabilities of the arm, shoulder, and hand; BP, bodily pain; MCS, mental component summary; OES, Oxford elbow score; PCS, physical component summary; PF, physical functioning; SF-36, Short Form-36; US, utility score; VAS, visual analog scale.

### Responsiveness

Longitudinal validity is shown in Table 3. The calculated Spearman’s rank correlation correlations were in line with predefined hypotheses in 36 out of the 42 (86%) values for the OES and 9 out of 12 (75%) for the *Quick*-DASH, indicating sufficient longitudinal validity for both instruments. Longitudinal validity was also sufficient for the OES subscales, with 83% (10/12), 75% (9/12), and 100% (12/12) hypotheses predicted correctly for the OES pain, function, and social-psychological subscale, respectively.

**Table 3:** Longitudinal validity of the instruments in patients with a simple elbow dislocation.

(Sub)scale		OES				*Quick*-DASH
		Pain	Function	Social- psychological	Total	
OES Pain	Pain	[1.00]	0.32*	0.45*	0.70*	0.45
Function	Function	0.32	[1.00]	0.53*	0.76*	**-0.68**
Social-psychological	Social-psychological	0.45	0.53	[1.00]	0.86*	-0.52
Total	Total	0.70	0.76	0.86	[1.00]	**-0.68**
*Quick*-DASH	Total	-0.45	**-0.68**	-0.52	**-0.68**	[1.00]
MEPI	Total	0.39	0.38	0.37	0.47	-0.57
Pain VAS	VAS	-0.37	-0.32	-0.32	-0.41	0.37
SF-36 PF	PF	**0.25**	0.51	0.41	0.50	**-0.63**
BP	BP	0.41	**0.24**	0.38	0.40	-0.41
PCS	PCS	**0.19**	0.39	0.39	0.38	-0.52
MCS	MCS	0.20	-0.003	0.05	0.11	0.005
EQ-5D US	US	0.36	0.47	0.42	0.50	-0.57
VAS	VAS	0.14	**0.35**	0.25	0.29	-0.18

N = 98 patients for all correlations except for the MEPI (N = 96) and EQ-5D VAS (N = 97). Rest of Table caption is identical to Table 2.

The standardized response mean (SRM) and the Effect Size (ES) of the OES and *Quick*-DASH instruments is shown in Table 4. As expected, the magnitude of change over time was large for the OES (sub)scales (SRM and ES >0.90). For the *Quick*-DASH, the SRM was large (0.87), but the ES was only moderate (0.73).

**Table 4:** Responsiveness: Standardized response mean (SRM) and effect size (ES) of the instruments in patients with a simple elbow dislocation.

(Sub)scale		N	Mean change	SD_change_	SRM	SD_6 weeks_	ES
OES	Total	98	25.2	15.6	1.61	18.5	1.36
	Pain	98	21.5	18.5	1.16	21.0	1.03
	Function	98	18.3	18.7	0.98	19.7	0.93
	Social-psychological	98	35.8	22.8	1.57	25.3	1.42
*Quick*-DASH	Total	98	-11.1	12.8	-0.87	15.1	-0.73
MEPI	Total	96	10.0	13.9	0.72	12.8	0.78
Pain	VAS	96	-0.73	1.63	-0.45	1.47	-0.50
SF-36	Total	98	7.16	10.43	0.69	9.60	0.75
	PF	98	3.19	7.79	0.41	7.64	0.42
	RP	98	15.32	13.10	1.17	10.96	1.40
	BP	98	8.01	8.65	0.93	7.08	1.13
	GH	98	0.03	7.70	0.004	8.51	0.004
	VT	98	0.71	8.93	0.08	7.57	0.09
	SF	98	2.91	10.94	0.27	8.99	0.32
	RE	98	1.85	9.76	0.19	8.94	0.21
	MH	98	-0.45	8.61	-0.05	7.62	-0.06
SF-36	PCS	98	9.02	8.31	1.09	7.84	1.15
SF-36	MCS	98	-1.87	10.08	-0.19	8.15	-0.23
EQ-5D	US	98	0.05	0.10	0.50	0.09	0.51
	VAS	97	2.2	11.1	0.20	11.3	0.20

Change scores were calculated from six weeks to 12 months.

*Quick*-DASH, *Quick* disabilities of the arm, shoulder, and hand; PF, physical functioning; RP, role limitations due to physical health; BP, bodily pain; ES, effect size; GH, general health perceptions; VT, vitality, energy, or fatigue; SF, social functioning; RE, role limitations due to emotional problems; MEPI, Mayo elbow performance index; MH, general mental health; OES, Oxford elbow score; PCS, physical component summary; MCS, mental component summary; SD, standard deviation (of mean change and of 6 weeks); SF-36, Short Form-36; SRM, standardized response mean.

### Floor and ceiling effects

**Fig 1:**
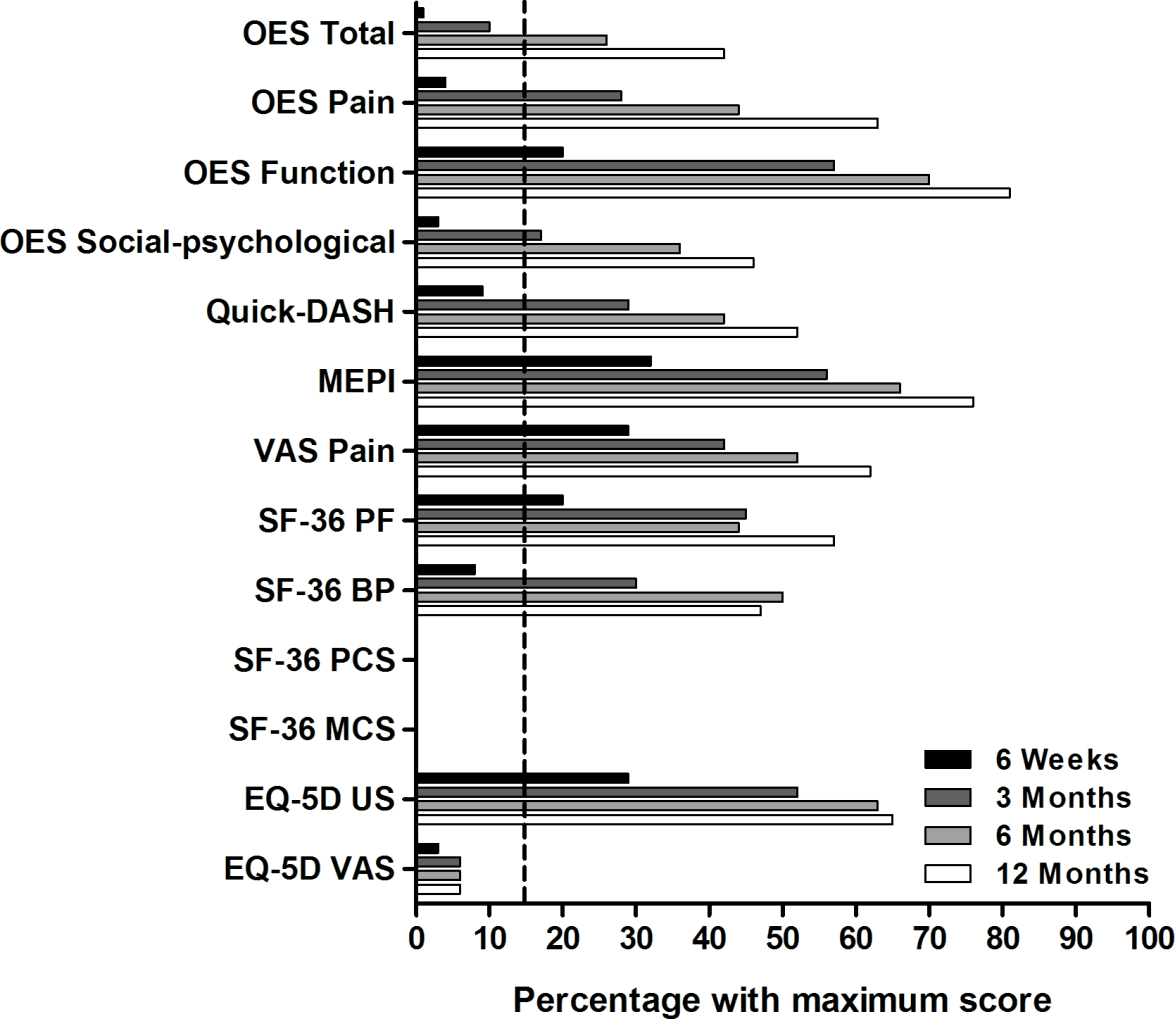
Ceiling effects of the instruments used in patients with a simple elbow dislocation. N = 99 for all (sub)scales at 6 weeks, N = 100 at 3 months (except for the MEPI (N = 99)), N = 97 at 6 months (except for the MEPI (N = 96)), and N = 99 at 12 months (except for the MEPI (N = 97) and EQ-5D VAS (N = 98)). The dotted line represents the acceptable 15% of patients with the maximum score. The SF-36 BP, PF, PCS and MCS did not demonstrate a ceiling effect and are not displayed. None of the (sub)scales demonstrated a floor effect.

None of the instruments evaluated showed a floor effect. From six weeks onwards the OES function, MEPI, VAS, SF-36 PF, and EQ-5D US demonstrated a ceiling effect (Fig 1); 20%, 32%, 29%, 20%, and 29% of the patients, respectively, reported the maximum score. From three months onwards the OES pain (28%) and social-psychological subscale (17%), *Quick-*DASH (29%), and SF-36 BP (30%) demonstrated a ceiling effect. The OES as a total scale demonstrated a ceiling effect only from six months onwards, where 26% of the patients reported the maximum score.

### Minimal important change and smallest detectable change

The number of patients per transition item for the different time intervals is shown in S2 Table. Anchor-based MIC and distribution-based SDC values are shown in Table 5. Overall, 57 transition items were reported as ‘slightly better’ and 31 as ‘no change’. The transition item demonstrated adequate correlation (*i*.*e*. r > 0.29) with the change scores of the OES total scale, the OES pain and function subscales, and the *Quick*-DASH. Spearman’s rank correlations with the transition item were below this threshold for the OES psychosocial subscale (r = -0.20) and all other (sub)scales. Therefore the MIC for the these could not be determined reliably.

**Table 5:** Minimal important change (MIC) and smallest detectable change (SDC) of the instruments in patients with a simple elbow dislocation.

(Sub)scale		Score range	MIC		SDC			
			N	MIC (95% CI)	N	SD_change_	SEM	SDC
OES	Total	0–100	57	8.2 (5.7–10.7)	31	6.1	4.3	12.0
	Pain	0–100	57	7.3 (3.3–11.4)	31	6.6	4.6	12.9
	Function	0–100	57	5.6 (2.0–9.2)	31	7.2	5.1	14.1
	Social-psychological	0–100	57	11.7 (7.6–15.9)	31	12.8	9.0	25.0
*Quick*-DASH	Total	0–100	57	3.5 (1.6–5.5)	31	6.2	4.4	12.2
MEPI	Total	0–100	56	N.A.	30	10.5	7.4	20.6
Pain	VAS	0–100	57	N.A.	31	0.9	0.6	1.7
SF-36	Total	0–100	57	N.A.	31	8.9	6.3	17.4
	PF	0–100	57	N.A.	31	4.7	3.4	9.3
	RP	0–100	57	N.A.	31	6.8	4.8	13.4
	BP	0–100	57	N.A.	31	5.9	4.2	11.6
	GH	0–100	57	N.A.	31	5.9	4.2	11.7
	VT	0–100	57	N.A.	31	7.9	5.6	15.6
	SF	0–100	57	N.A.	31	10.5	7.4	20.5
	RE	0–100	57	N.A.	31	10.7	7.5	20.9
	MH	0–100	57	N.A.	31	7.3	5.1	14.3
SF-36	PCS	0–100	57	N.A.	31	5.4	3.8	10.5
SF-36	MCS	0–100	57	N.A.	31	9.6	6.8	18.8
EQ-5D	US	0–1	57	N.A.	31	0.1	0.04	0.1
	VAS	0–100	57	N.A.	31	10.1	7.1	19.8

Anchor-based and distribution-based methods for Minimal Important Change (MIC) and Smallest Detectable Change (SDC) values, respectively. Change scores were calculated from six weeks to 12 months. MIC is the mean score for patients who reported ‘slightly better’ on the transition item. It is shown with the 95% confidence interval between brackets.

N.A., not applicable, as the correlation with the transition item was <0.29.

MIC, minimal important change; SD_change_, standard deviation of the change score of patients that reported ‘no change’ on the transition item; SEM, standard error of measurement; SDC, smallest detectable change.

For the OES, the anchor-based MIC was 8.2 points (95% CI 5.7–10.7) for the total scale, 7.3 (95% CI 3.3–11.4) points for the pain subscale, 5.6 (95% CI 2.0–9.2) points for the function subscale, and 11.7 (95% CI 7.6–15.9) points for the social-psychological subscale (Table 5). The anchor-based MIC for the *Quick*-DASH change score was 3.5 (95% CI 1.6–5.5) points. The ROC curve analysis produced similar results, with wider confidence intervals. There the MIC was 6.3 (95% CI 4.2–8.3) points for the OES and 4.5 (95% CI 2.3–11.4) for the *Quick*-DASH.

For each of these four (sub)scales, the MIC was smaller than the SDC values. These SDC was 12.0 (SEM 4.3) for the OES total scale, 12.9 (SEM 4.6) for the OES pain subscale, 14.1 (SEM 5.1) for the OES function subscale, 25.0 (SEM 9.0) for the OES social-physiologic subscale, and 12.2 (SEM 4.4) for the *Quick*-DASH.

## Discussion

This study showed that the OES and *Quick*-DASH are reliable, valid, and responsive instruments for the evaluation and follow-up of patients after a simple elbow dislocation that was treated non-operatively. The anchor-based MIC was 8.2 points for OES and 3.5 for *Quick*-DASH.

The reliability of the OES (Cronbach’s alpha 0.882) and *Quick*-DASH (Cronbach’s alpha 0.886) was comparable with published values [10, 13, 23, 25, 40–44]. The OES has previously been acknowledged as the most reliable questionnaire [8]. The current data confirm that it is at least as good as the DASH. The MEPI demonstrated inadequate internal consistency which had also been shown previously [45].

The OES proved its validity by demonstrating strong correlations with the *Quick*-DASH and SF-36 BP and PCS. The latter is a novel observation, as no data were available on the correlation between the OES and SF-36 subscales. Correlation with the *Quick*-DASH and MEPI has been published before for patients who had undergone elbow surgery [9, 15].

There is no available literature concerning the validity of the OES and *Quick*-DASH in non-operatively treated patients with an elbow dislocation. Construct validity of the (*Quick*-)DASH has been reported before [40, 46]. The correlation in change scores between the subdomains of the OES and *Quick*-DASH are comparable with data from Dawson *et al*. [9]. Change scores of the OES correlated moderately with change scores of the *Quick*-DASH and MEPI. The moderate correlation of change scores of the OES and MEPI could be explained by the fact that the MEPI demonstrated significant ceiling effects from the first follow-up onwards. The ceiling effect does not allow to detect actual changes over time.

The finding that the standardized response mean (SRM) and effect size (ES) of the OES and *Quick*-DASH (sub)scales were large (except moderate ES for *Quick*-DASH) suggests that both instruments display good to excellent ability to detect clinical change over time. Moderate to large ES and large SRM values have been shown before for the (*Quick*-)DASH or DASH [25, 40, 47–49].

All instruments displayed a ceiling effect. This was as expected, since the type of elbow dislocations studied are relatively mild injuries, with expected full recovery within six months. Full recovery implies the largest score, and hence a ceiling effect. A similar phenomenon was also seen for the DASH in patients treated for a humeral shaft fracture [50]. Patients treated operatively for Dupuytren’s contracture also showed ceiling effect for the DASH from three months after surgery onwards [51]. The expected ceiling effect is not a problem per se, but one should realize that the instruments are not useful for comparing treatment outcome at times where a ceiling effect is observed.

The interpretability represented by the MIC was 8.2 for the OES total score. The MIC for the OES pain, function and social-psychological subdomains (7.3, 5.6, and 11.7 points, respectively) were lower than for patients who underwent elbow surgery for chronic elbow pathologies (17.41–19.23, 9.23–9.64, and 17.79–18.30 points, respectively) as reported before [9]. Fourteen patients in their study answered ‘slightly better’ on the transition item, which was much lower than the 57 patients in the current study. The difference in population (and recovery pattern) most likely explains the difference in MIC [4]. Patients with chronic pathology like in Dawson’s study have a moderate to poor score at baseline and retain functional limitation after surgery. The patients in the current study with an acute injury started at full loss of function immediately after injury, and the majority showed full recovery at the end. MIC values are known to differ depending on patient population and the type of injury and intervention [9, 37]. Although the MIC for the OES in this study were evaluated in a cohort of patients with a simple elbow dislocation, one may expect that the MIC can be extrapolated to also be useful in the evaluation of other acute elbow injuries where full recovery is to be expected.

The MIC for the *Quick-*DASH was only 3.5 points. This is hard to believe for a scale that runs from 0 to 100, especially as previously published anchor-based MIC values for the (*Quick*-)DASH-score ranged from 8 to 19 points [47, 48, 52–55]. Data on patients with an elbow dislocation are not available. The most plausible explanation for this is again the fact that already on the first evaluation (six weeks) the *Quick-*DASH showed a ceiling effect, which implies that subtle impediments and changes cannot be measured from that point onward. This emphasizes the need for elbow-specific questionnaires like the OES for the less severe types of injuries. The OES also demonstrated a ceiling effect, however not before the six months follow-up moment, at which time patients were recovered to the largest degree.

Ideally, the MIC should be larger than the smallest detectable change (SDC) in order to be able to differentiate between ‘real’ change and change caused by measurement error [4]. For the OES and *Quick*-DASH the SDC was larger than the MIC. For the *Quick*-DASH, Polson *et al*. reported a SDC of 11, which was lower than 19 for the MIC [47]. For the OES, a previous study that also used both anchor- and distribution-based methods for calculating the MIC, also found that SDC values were higher than anchor-based MIC values [9]. Our findings confirm this. It implies that any change score reported by a patient that is larger than the MIC but smaller than the SDC should be interpreted with care; it may represent clinical improvement but can also be due to chance.

The SEM in the current study was calculated with the corresponding change scores of patients that answered ‘no change’ on the transition item as a surrogate for test-retest values. This could have introduced some bias, which might have influenced the SDC value. Future studies should include an adequate test-retest analysis in order to be able to calculate a true SEM. Nevertheless, the anchor-based MIC values are the closest estimate of actual clinical change, therefore the MIC values in current study are of definite value.

This study has some limitations. First, the relatively long time between the follow-up moments hindered an adequate test-retest analysis. Furthermore, it could also have led to recall bias with regard to the transition item. However, the interval for the transition item in the only other study that analyzed the MIC of the OES using an anchor-based approach was at least six months [9]. Secondly, the transition item for the MIC analysis included “completely recovered” was a heterogeneous group. This group included patients who 1) were already completely recovered at the previous follow-up visit; 2) truly experienced no change; or 3) reported complete recovery for the first time but actually improved little/much since the previous follow-up. For future studies the outlying answers (*i*.*e*., *“*completely recovered” and “worse than ever”) should be left out. Finally, there were insufficient data for evaluating whether the MIC values were the same for the consecutive time intervals.

Strengths of this study were its sample size and homogenous patient population. Furthermore, to the best of our knowledge, it is the first study to validate the OES for patients with elbow injuries treated non-operatively. Previous studies focused primarily on operated patients [9, 10, 13, 14, 23].

## Conclusion

The OES and *Quick*-DASH have proven to be reliable, valid, and responsive instruments for evaluating elbow-related quality of life in patients who sustained a simple elbow dislocation. Whereas validity of the OES was known for surgically treated chronic elbow pathologies, this study demonstrated the OES is also valid for acute elbow injuries treated non-operatively. Both instruments are useful for research purposes, and could play an important role in daily practice. The MIC and SDC values facilitate statistical power analysis and sample-size calculations for future clinical studies.

## Supporting information

S1 Table **Hypothesized correlations between the instruments for (A) construct validity and (B) Longitudinal validity in patients with a simple elbow dislocation**.
Expected strength of correlation or all possible combinations; r>0.6 indicates high correlation, 0.3<r>0.6 moderate correlation, and r> 0.6 low correlation.
*Quick*-DASH, *Quick* disabilities of the arm, shoulder, and hand; BP, bodily pain; MCS, mental component summary; OES, Oxford elbow score; PCS, physical component summary; PF, physical functioning; SF-36, Short Form-36; US, utility score; VAS, visual analog scale.
(PDF)

S2 Table Answers to the transition item at three different time windows in patients with a simple elbow dislocation. Data are shown as number with valid percentage.
(PDF)
