# Supplementary material for: Minimal important change and other measurement properties of the Oxford Elbow Score and the Quick Disabilities of the Arm, Shoulder, and Hand in patients with a simple elbow dislocation; validation study alongside the multicenter FuncSiE trial
Source: PLoS One. 2017 Sep 8;12(9):e0182557. doi: 10.1371/journal.pone.0182557 (PMC5590744; doi:10.1371/journal.pone.0182557)
Supplement: S1 Table — Hypothesized correlations between the instruments for (A) construct validity and (B) Longitudinal validity in patients with a simple elbow dislocation. Expected strength of correlation or all possible combinations; r>0.6 indicates high correlation, 0.30.6 moderate correlation, and r> 0.6 low correlation. Quick-DASH, Quick disabilities of the arm, shoulder, and hand; BP, bodily pain; MCS, mental component summary; OES, Oxford elbow score; PCS, physical component summary; PF, physical functioning; SF-36, Short Form-36; US, utility score; VAS, visual analog scale. (PDF) [file pone.0182557.s001.pdf]

S1 Table. Hypothesized correlations between the instruments for (A) construct validity and (B) Longitudinal validity in patients with a simple elbow dislocation

A. Construct validity

**(Sub)scale**		**OES**				***Quick*-DASH**
		**Pain**	**Function**	**Social-psychological**	**Total**	
OES	Pain	[N.A.]	moderate*	moderate*	high*	high
	Function	moderate	[N.A.]	moderate*	high*	high
	Social-psychological	moderate	moderate	[N.A.]	high*	high
	Total	High	high	high	[N.A.]	high
Quick-DASH		High	high	high	high	[N.A.]
MEPI		moderate	moderate	moderate	moderate	high
Pain	VAS	moderate**	moderate	moderate	moderate	moderate
SF-36	PF	moderate	moderate	moderate	moderate	high
	BP	High	high	high	high	high
	PCS	moderate	moderate	moderate	moderate	high
	MCS	Low	low	low	low	low
EQ-5D	US	moderate	moderate	moderate	moderate	moderate
	VAS	Low	low	low	low	low

B. Longitudinal validity

(Sub)scale		OES				*Quick*-DASH
		**Pain**	**Function**	**Social-psychological**	**Total**	
OES	Pain	[N.A.]	moderate*	moderate*	high*	moderate
	Function	moderate	[N.A.]	moderate*	high*	moderate
	Social-psychological	moderate	moderate	[N.A.]	high*	moderate
	Total	high	high	high	[N.A.]	moderate
Quick-DASH		moderate	moderate	moderate	moderate	[N.A.]
MEPI		moderate	moderate	moderate	moderate	moderate
Pain	VAS	moderate	moderate	moderate	moderate	moderate
SF-36	PF	moderate	moderate	moderate	moderate	moderate
	BP	moderate	moderate	moderate	moderate	moderate
	PCS	moderate	moderate	moderate	moderate	moderate
	MCS	low	low	low	low	low
EQ-5D	US	moderate	moderate	moderate	moderate	moderate
	VAS	low	low	low	low	low

Expected strength of correlation or all possible combinations; r>0.6 indicates high correlation, 0.3<r>0.6 moderate correlation, and r> 0.6 low correlation.

Quick-DASH, *Quick* disabilities of the arm, shoulder, and hand; BP, bodily pain; MCS, mental component summary; OES, Oxford elbow score; PCS , physical component summary; PF, physical functioning; SF-36, Short Form-36; US, utility score; VAS, visual analog scale.

* Correlations between brackets are self-correlations and are not included in the calculation of the percentage correlations predicted correctly. For the OES, the overall number of correlations is 42 (the six correlations given above the self-correlation that are marked with an asterisk are also mentioned in the columns to the left as reversed correlation, and are thus superfluous).

** A moderate correlation was expected, since we know from experience that patients report a larger variety in scores on a visual scale than when they are asked to choose between five discrete options. The OES Pain covers pain over the last four weeks and is a combination of five questions. Only one of these questions asks for the amount of pain (like in the VAS), and the

other four ask about the extent to which pain has influenced their life. The VAS on the other hand, is a single question asking for the amount of pain during the last week.
