# Supplementary material for: Minimal important change and other measurement properties of the Oxford Elbow Score and the Quick Disabilities of the Arm, Shoulder, and Hand in patients with a simple elbow dislocation; validation study alongside the multicenter FuncSiE trial
Source: PLoS One. 2017 Sep 8;12(9):e0182557. doi: 10.1371/journal.pone.0182557 (PMC5590744; doi:10.1371/journal.pone.0182557)
Supplement: S2 Table — Data are shown as number with valid percentage. (PDF) [file pone.0182557.s002.pdf]

S2 Table. Answers to the transition item at three different time windows in patients with a simple elbow dislocation

Transition item	Total	Time window		
		6 weeks to 3 months	3 to 6 months	6 to 12 months
1 'completely recovered'	69 (29%)	11 (16%)	24 (30%)	34 (38%)
2 'much better'	74 (31%)	36 (51%)	25 (32%)	13 (14%)
3 'slightly better'	57 (24%)	17 (24%)	19 (24%)	21 (23%)
4 'no change'	31 (13%)	5 (7%)	9 (11%)	17 (19%)
5 'slightly worse'	9 (4%)	2 (3%)	2 (3%)	5 (6%)
6 'much worse'	0 (0%)	0 (0%)	0 (0%)	0 (0%)
7 'worse than ever	0 (0%)	0 (0%)	0 (0%)	0 (0%)
Missing data	60	29	21	10

Data are shown as number with valid percentage.
